# Angiogenesis: An Adaptive Dynamic Biological Patterning Problem

**DOI:** 10.1371/journal.pcbi.1002983

**Published:** 2013-03-21

**Authors:** Timothy W. Secomb, Jonathan P. Alberding, Richard Hsu, Mark W. Dewhirst, Axel R. Pries

**Affiliations:** 1 Department of Physiology and Arizona Research Laboratories, University of Arizona, Tucson, Arizona, United States of America; 2 Department of Radiation Oncology, Duke University Medical Center, Durham, North Carolina, United States of America; 3 Charité - Universitätsmedizin Berlin, Department of Physiology and CCR, Berlin, Germany; University of Kansas Medical Center, United States of America

## Abstract

Formation of functionally adequate vascular networks by angiogenesis presents a problem in biological patterning. Generated without predetermined spatial patterns, networks must develop hierarchical tree-like structures for efficient convective transport over large distances, combined with dense space-filling meshes for short diffusion distances to every point in the tissue. Moreover, networks must be capable of restructuring in response to changing functional demands without interruption of blood flow. Here, theoretical simulations based on experimental data are used to demonstrate that this patterning problem can be solved through over-abundant stochastic generation of vessels in response to a growth factor generated in hypoxic tissue regions, in parallel with refinement by structural adaptation and pruning. Essential biological mechanisms for generation of adequate and efficient vascular patterns are identified and impairments in vascular properties resulting from defects in these mechanisms are predicted. The results provide a framework for understanding vascular network formation in normal or pathological conditions and for predicting effects of therapies targeting angiogenesis.

## Author Summary

The blood vessels provide an efficient system for transport of substances to all parts of the body. They are capable of growing or regressing during development, in response to changing functional needs, and in disease states. This is achieved by structural adaptation, i.e. changes in the diameters and other characteristics of existing vessels, and by angiogenesis, i.e. growth of new blood vessels. Here, we address the question: How do the processes of structural adaptation and angiogenesis lead to the formation of organized vessel networks that can supply the changing needs of the tissue? We carried out theoretical simulations of network growth and adaptation, including vessel blood flows, oxygen transport to tissue, and the generation of a growth factor in low-oxygen regions, which stimulates angiogenesis by sprouting from existing vessels. We showed that the processes of over-abundant random angiogenesis together with structural adaptation including pruning of redundant vessels can generate adequate and efficient vessel networks that are capable of continuously adapting to changing tissue needs. Our work provides insight into the biological mechanisms that are essential for formation and maintenance of functional vessel networks, and may lead to new strategies for controlling blood vessel formation in diseases.

## Introduction

Vascular systems develop, adapt and remodel in response to local and systemic needs [1]. Over hours to days, blood vessels form and grow (vasculogenesis and angiogenesis), undergo structural adaptation (remodeling), or regress (pruning) [2]. These processes of vascular network patterning or angioadaptation [3] are essential for many functions of the circulatory system, including growth, responses to sustained exercise, estrus cycle, pregnancy, wound healing and ageing. Furthermore, they are centrally involved in diseases including hypertension, tissue ischemia (coronary heart disease, stroke) and tumor growth, and in natural and therapeutic responses to these diseases.

**Figure 1:**
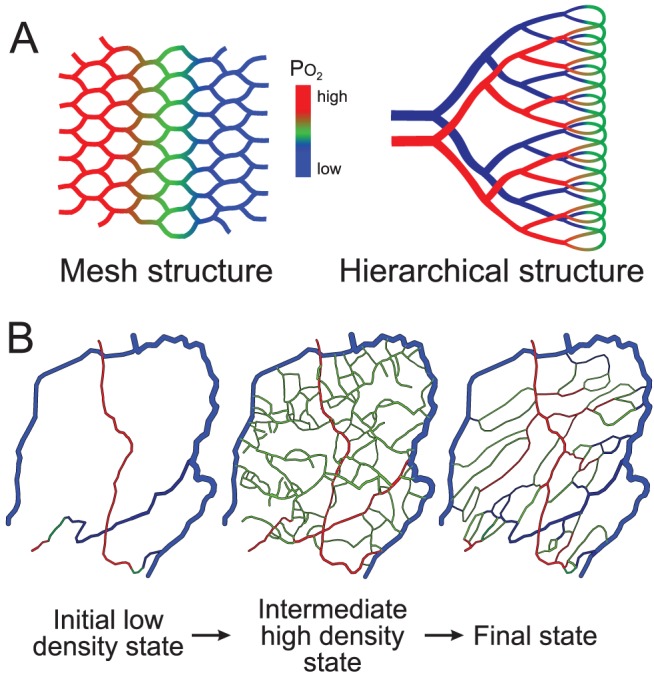
Concepts of microvascular pattern formation. (**A**) Microvascular networks are often conceptualized as mesh or hierarchical structures. A mesh minimizes diffusion distances between capillaries and tissue but has high flow resistance and results in non-uniform oxygen levels from the arterial to the venous side. In a hierarchical structure, larger supply vessels decrease flow resistance, but regions surrounding those vessels are inadequately supplied due to large diffusion distances. Colors (red - green - blue) indicate flow from arterial to venous vessels, with decline in oxygen levels. (**B**) Hypothesized steps in generation of functional vascular networks. A dense network of vessels is generated by over-abundant angiogenesis and refined by structural adaptation and pruning. Resulting networks combine features of mesh and hierarchical structures.

To function efficiently, vascular networks must satisfy two apparently conflicting requirements. The demand for oxygen and its low solubility in tissue necessitate a dense network, such that its distance from tissue cells does not exceed the maximum oxygen diffusion distance (about 30 µm in the heart). A dense mesh-like structure can satisfy this requirement, but at the expense of high resistance to flow (Figure 1A). Conversely, a hierarchical tree-like structure can deliver flow to terminal branches efficiently but does not provide a spatially uniform vascular supply. Actual microvascular networks combine both types of structures: a hierarchical system is embedded in the supplied region so that exchange vessels are distributed with approximately uniform density. A further requirement is the ability to adapt to varying demands, while maintaining flow. Thus, the development of vascular networks presents a complex problem of biological pattern formation.

How is this patterning problem solved? Much research has focused on molecular and cellular aspects of angiogenesis and anti-angiogenesis and on translating the results to the clinic for the treatment of hypoxic conditions (e.g. vascular occlusion) or unwanted vascularization (e.g. tumor growth). However, the formation of functional vascular networks remains poorly understood. In early development, before circulation starts, vascular patterning is genetically determined [4], but genetic information cannot specify the individual positions and behavior of more than 10^9^ vessels in the human body. We hypothesize that the problem of vascular patterning is ‘solved’ by stochastic sprouting angiogenesis in response to a growth factor generated in hypoxic regions (e.g. vascular endothelial growth factor, VEGF), coupled to structural reactions (growth, regression, elimination) of each vessel to mechanical and biochemical stimuli. According to this hypothesis, angiogenesis results in networks with disordered structures, which organize themselves into functional networks through structural adaptation and pruning [2] (Figure 1B).

To test this hypothesis and analyze the relations between biological mechanisms and system properties, we developed a theoretical model that integrates simulations of network blood flow, convective and diffusive oxygen transport, generation and diffusion of VEGF [5], stochastic sprouting angiogenesis [6], structural adaptation and vessel elimination by pruning [3]. Several theoretical models for angiogenesis have been developed [7]–[18]. Our approach combines elements of those studies with a model for structural adaptation of vessel diameters [19], [20] including information transfer by conducted responses along vessel walls, which is needed for proper flow distribution and avoidance of functional shunts [21].

The model is based on experimental observations of network structure and hemodynamics in rat mesentery, a thin sheet-like tissue [22]. In brief, VEGF is generated in hypoxic regions [23]. Diffusive transport of oxygen and VEGF is simulated [24]. Vessel sprouts are generated with a VEGF-dependent probability. Non-flowing sprouts maintain a fixed diameter [25] until they connect to other segments and commence flow. Diameters of flowing segments vary with time according to generic rules, including responses to mechanical stimuli (intravascular pressure and wall shear stress) and to metabolic status (represented by intravascular oxygen partial pressure, *P_O_*_2_). Vessels are pruned if their diameter falls below a critical threshold. Under these assumptions, the model predicts the time-dependent development of the network structure, including the positions, lengths and diameters of each segment, and the resulting distributions of blood flow, oxygen and VEGF.

## Methods

### Observations of mesenteric network

**Figure 2:**
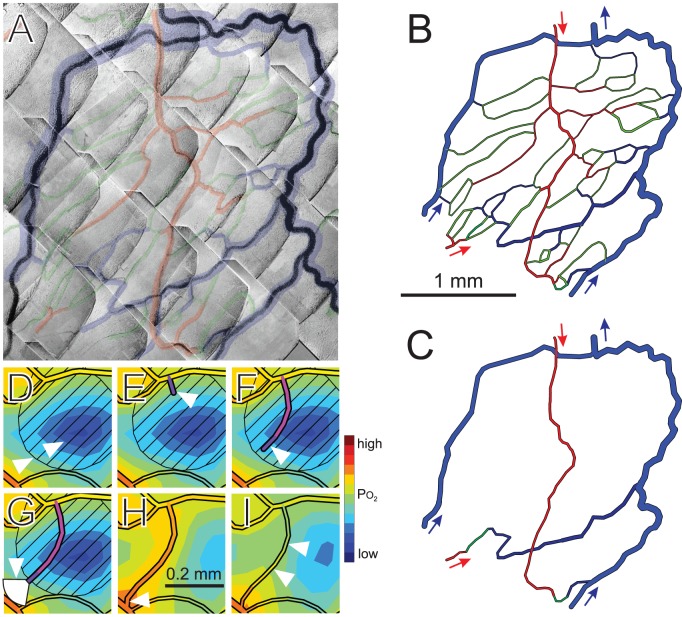
Steps in simulation approach. (**A**) Network of microvessels in rat mesentery, imaged using intravital microscopy. Shaded overlay highlights vessel positions, with arterioles (red), capillaries (green) and venules (blue). This region was selected for analysis because the outer loop of venules provides stable boundary conditions for the tissue domain. (**B**) Computer generated image of network structure, trimmed to reduce the number of network boundary nodes to five (arrows). (**C**) Network skeleton, used as initial condition for simulations. In (**D–I**), a small region within a typical simulation is shown at a sequence of times indicating aspects of the method. White triangles denote features mentioned in this caption. (**D**) The oxygen field surrounding the vessels is computed using the Green's function method. Blue shades denote low oxygen levels. VEGF is assumed to be generated in hypoxic regions and to diffuse according to local gradients, and the resulting VEGF field is computed. Diagonal hatching indicates VEGF concentration above a given threshold. (**E**) On vessels lying in regions with VEGF above threshold, sprouts are generated with probability dependent on local VEGF concentration. (**F**) A fixed rate of sprout elongation is assumed. Direction of growth is randomly varied at each time step. (**G**) If other vessels lie within a sector of radius 100 µm ahead of the sprout tip, the growth is biased towards them. (**H**) A sprout reaching another vessel forms a connection, allowing flow. (**I**) Diameters of flowing vessels adapt to metabolic and hemodynamic stimuli.

In procedures approved by the University and State authorities for animal welfare, the small bowel of a male wistar rat was exteriorized and a fat-free portion of the mesenteric vascular network was observed by intravital microscopy [22]. Papaverine (10^−4^ M) was continuously applied to suppress vessel tone. The spatial arrangement and diameters and lengths of segments were measured (Figure 2A), together with hematocrit and blood flow velocity for vessels entering and leaving the network [26], [27].

### Overview of model

The theoretical model combines a network-oriented analysis of blood flow, angiogenesis and structural adaptation with a continuum analysis of oxygen and VEGF delivery, production, diffusion and uptake. The network is represented as a set of straight segments with defined positions, diameters and blood flow rates. The simulation is implemented using the C language on personal computers. Typical run time is about 1 minute per time step. Parameter values are given in Table 1. The precise values are stated for reproducibility, but the number of decimals shown does not imply a corresponding precision in their estimation.

**Table 1:** Model parameters.

**Blood parameters**		
Maximal RBC oxygen concentration	*C*_0_ = 0.5 cm^3^O_2_ cm^−3^	[24]
Effective unbound oxygen solubility	α*_eff_* = 3.1×10^−5^ cm^3^O_2_ cm^−3^ mmHg^−1^	[24]
Hill equation parameter	*P*_50_ = 38 mmHg	[24]
Hill equation parameter	*n* = 3	[24]
**Tissue oxygen parameters**		
Krogh diffusion constant	*D_O_* α = 6×10^−10^ cm^3^O_2_ cm^−1^ s^−1^ mmHg^−1^	[24]
Consumption rate	*M*_0_ = 0.5–2.5 cm^3^O_2_ (100 cm^3^)^−1^ min^−1^	See text
*P_O_*_2_ at half-maximal consumption	*P_c_* = 1 mmHg	[24]
**VEGF parameters**		
Diffusivity	*D_G_* = 1.13×10^−6^ cm^2^ s^−1^	[51]
Basal release rate	*M_G_*_0_ = 1.97×10^−3^ pM s^−1^	[51]
Tissue degradation rate constant	*K_G_* = 2.82×10^−3^ s^−1^	See text
**Structural adaptation parameters**		
Reference wall shear stress	*τ_ref_* = 0.103 dyn/cm^2^	[20]
Reference oxygen level for metabolic signal	*P_O_*_2*ref*_ = 93.2 mmHg	[20]
Conducted response length constant	*L_c_* = 17300 µm	[20]
Reference flow rate for metabolic signal	*Q_ref_* = 0.198 nl/min	[20]
Conducted response saturation	*J*_01_ = 1000 µm	See text
Pressure sensitivity	*k_p_* = 0.68	[20]
Metabolic sensitivity	*k_p_* = 0.70	[20]
Conducted response sensitivity	*k_c_* = 2.45	[20]
Shrinking tendency	*k_s_* = 2.549	See text
Randomization of shrinking tendency	*Ran-k_s_* = 0.1	[47]
Structural adaptation time scale	*T* = 4.5 day	[46]
**Angiogenesis parameters**		
Time step	Δ*t* = 1 day	
Diameter of new sprouts	*D_s_* = 10 µm	[25]
Threshold VEGF concentration	*C_th_* = 0.8 nM	See text
Constant in sprouting probability function	*C_th_*_50_ = 0.5 nM	See text
Maximum sprout formation probability	*k_p_* = 0.002 µm^−1^ day^−1^	[12]
Sprout growth rate	*V_g_* = 50 µm day^−1^	[39]
Attraction constant to nearby vessels	*k_V_* = 10 µm^−1^	See text
Maximum vessel sensing distance	*R_max_* = 100 µm	[41]
Maximum vessel sensing angle	θ*_max_* = π/3	See text
Variance of growth direction randomization	σ_s_ = 0.1	See text
**Vessel migration parameters**		
Threshold for migration	*λ_t_* = 0.05	See text
Maximum migration velocity	*v_max_* = 1 µm day^−1^	See text

### Initial network structure

To create an initial condition, the network was reduced to a minimal ‘skeleton,’ retaining five boundary nodes at which blood flows enters or exits the network (Figure 2C). The network lies in a thin (20 µm) sheet of tissue with area 4.23 mm^2^. Flow or pressure conditions in these segments were specified based on simulations of a larger network containing the region [28], and are typical for vessels of these sizes and types in this tissue. Two arterioles feed the region with a *P_O_*_2_ of 75 mmHg. One arteriole (upper in Figure 2C) is held at a fixed pressure of 59.09 mmHg with an inflow hematocrit of 0.3742. The flow rate in the other feeding arteriole is 15 nl/min. Two venules flow into the network and form boundaries for the tissue domain. Each venule has a flow rate of 28.1 nl/min, an inflow hematocrit of 0.4 and a *P_O_*_2_ of 38 mmHg. The diameters of these venules are fixed to provide stable conditions on the boundary of the tissue domain. The venules converge at a single outflow, which is assigned a pressure of 15 mmHg.

### Flow rates

The method for simulating blood flow in microvascular networks follows established approaches [28], [29]. The network is represented as a set of resistive elements meeting at nodes. The flow resistance of a segment is
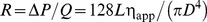
(1)where *L* and *D* are length and diameter, Δ*P* is pressure drop and η_app_ is apparent viscosity of blood, dependent on diameter and hematocrit [28], [30]. Non-uniform partition of hematocrit at diverging bifurcations is included [30], [31]. The flows into each node are expressed in terms of nodal pressures and flow resistances. Setting the sum of flows to zero gives a system of linear equations for nodal pressures [32], which is solved iteratively. The wall shear stress is
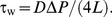
(2)


Because flow resistance depends on hematocrit, a further iterative process is required, in which hematocrits are recalculated, resistances are updated and flows are recomputed. This is repeated until changes in flows and hematocrits do not exceed a small tolerance.

### Oxygen field

The physical principles governing convective and diffusive transport in tissue are well established [33]. The steady-state distribution of oxygen in vessels and tissue is computed using a two-dimensional implementation of the computationally efficient Green's function method [24], [34]. The partial pressure of oxygen *P_O_*_2_(*x*,*y*) satisfies
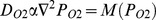
(3)where *D_O_*_2_ and α are diffusivity and solubility in tissue. The oxygen consumption rate *M*(*P_O_*_2_) is governed by Michaelis-Menten kinetics
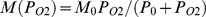
(4)where *M*_0_ represents demand, assumed uniform, and *P*_0_ represents *P_O_*_2_ at half-maximal consumption. Convective oxygen flux in blood is
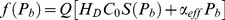
(5)where *Q* is flow rate, *H_D_* is discharge hematocrit,
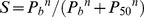
(6)is oxyhemoglobin saturation, *P_b_* is blood *P_O_*_2_, *C_0_* is oxygen-binding capacity of red blood cells, *P*_50_ is *P_O_*_2_ at 50% saturation, *n* is a constant and α*_eff_* is effective solubility of oxygen in blood. The boundary conditions are continuity of *P_O_*_2_ and oxygen flux at the blood-tissue interface. The oxygen field is expressed as a superposition of fields resulting from an array of sources (representing vessels) and sinks (representing tissue regions), whose strengths are computed so as to match intravascular and extravascular oxygen levels. The sinks are located on a square array of tissue points spaced 50 µm apart throughout the region spanned by the network. Effects of intravascular resistance to radial oxygen diffusion [35] are included. Very short segments compromise the numerical stability of this method for solving convection-diffusion problems. Simulations were designed such that segments have a minimum length 10 µm.

### Growth factor field

Among the multiple chemical factors that influence the formation and growth of blood vessels, VEGF plays a key role [36]. Released in hypoxic regions, it stimulates the growth of new vessels, which may increase oxygen supply to those regions. In the present model, this process is simulated based on previously developed models for spatial distribution of VEGF in skeletal muscle tissue [5], [23]. VEGF is released by parenchymal cells at a rate that depends on *P_O_*_2_, diffuses through tissue with diffusivity *D_G_*, and is degraded or taken up uniformly with linear kinetics and rate constant *K_G_*. Its concentration *C_G_*(*x*,*y*) satisfies
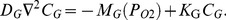
(7)


The dependence of release rate on *P_O_*_2_ (in mmHg) is:
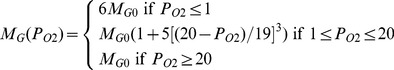
(8)where *M_G_*_0_ is the basal rate. These equations were developed by Ji et al. [23]. The value of *K_G_* was estimated as follows. A typical length scale for concentration gradients is *L_diff_*  =  (*D_G_*/*K_G_*)^1/2^. The results of Ji et al. [23] imply that *L_diff_*≈200 µm, so *K_G_* = 2.82×10^−3^ s^−1^. Resulting values of *C_G_* are in the range *M_G_*_0_/*K_G_* to 6*M_G_*_0_/*K_G_*, i.e. 0.7 to 4.2 pM, consistent with the results of Ji et al. *C_G_*(*x*,*y*) is computed using the Green's function method, neglecting exchange of VEGF between tissue and vessels.

### Sprout formation

Angiogenesis is assumed to occur by sprouting from existing vessels [37]. Splitting angiogenesis (intussusception) is not included. Numerous models for sprouting angiogenesis have been proposed. The present approach follows that of [12]. At each time step, a point is selected with uniform probability on each segment, local *C_G_* is computed and a sprout is formed with probability
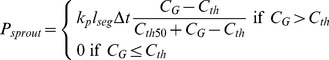
(9)where *k_p_* is the maximal probability of sprout formation per length per time. This functional dependence is chosen to give threshold concentration *C_th_* for sprout formation and approach to the maximal probability at large concentrations [12]. If a sprout forms within 10 µm of a node on the parent segment, it is moved to that node. If the sprout is at a network boundary node or an existing branch point, it is suppressed. These rules were introduced for technical reasons as already mentioned, and do not substantially affect the patterning process.

In this two-dimensional implementation, the sprout direction is randomly ±90° to the parent segment. Sprouts maintain a diameter of *D_s_* = 10 µm [25] until they become part of a flow pathway, and are subject to structural adaptation. The threshold VEGF concentration *C_th_* is a critical parameter. A low value gives uncontrolled angiogenesis and network instability. A high value gives inadequate vascular density. The chosen value *C_th_* = 0.8 pM gives adequate, stable network structures over a range of oxygen demand, lies between the values observed experimentally at rest and in exercise [38], and is within the range predicted by theoretical models [5], [23]. The chosen value of *C_th_*_50_, 0.5 pM, gives rapid approach to the maximal rate of sprout formation as *C_G_* increases.

### Elongation of sprouts

The simulation of sprout growth follows previous work [12]. Sprouts are assumed to elongate at constant rate *V_g_* until they connect with another vessel. Reported growth rates vary; *V_g_* = 50 µm/day is assumed [39]. The direction of endothelial cell migration shows persistence with time [40]. To represent effects of heterogeneity in extracellular matrix structure, the current direction ***d*** is rotated by a random angle from a Gaussian distribution with zero mean and variance σ_s_, giving a direction ***d*** *′* for the next time step. This variance gives vessel tortuosity consistent with that seen in mesenteric networks.

The tip cells leading the growth of endothelial sprouts possess filopodia, elongated processes that explore the tissue for distances of up to 100 µm [41], and may allow the sprout to sense other vessels. Such a homing mechanism, which was not included in previous models [12], is needed since otherwise sprouts in three-dimensional tissues would rarely intersect other vessels. In the model, sprouts are attracted by other vessels lying within a sector extending a distance *R_max_* from the tip and an angle *θ_max_* from the previous growth direction. The attraction decreases with distance *r* from the tip, and with angle *θ* from the previous growth direction. The vector sum
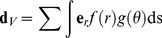
(10)is constructed, where the sum is over the segments within the sector, the integral is along each segment, and
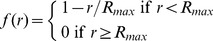
(11)

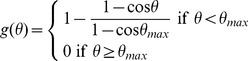
(12)


The new sprout direction is
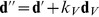
(13)where *k_V_* represents sensitivity of growth direction to existing vessels. The functions introduced in equations (11) and (12) are chosen so that the effect of other vessels on sprout growth falls to zero at the edge of the sector explored by filopodia. The specific forms of these functions are not important. The results are, however, sensitive to the assumed values of the sensing radius *R_v_* and the sensitivity *k_v_*. The sensing radius is set equal to the maximum observed length of filopodia, 100 µm [41]. A large value of *k_V_* is chosen, so that vessels are strongly directed toward vessels within the sensing radius.

At each time step, all sprouts are elongated by *V_g_*Δ*t* in increments of 5 µm. If the distance of a tip to any other segment is less than 5 µm, a new segment is created linking the tip to the nearest point on that segment. If necessary, the resulting intercept point is moved to eliminate short segments (<10 µm). If the intercept point is at a network boundary node or if the segment intersects the boundary of the tissue domain, the sprout is suppressed. These rules were introduced for technical reasons, and do not substantially affect the patterning process.

In previous models, sprout growth was biased up the gradient of VEGF concentration [12]. Here, it was found that this interferes with formation of new flow pathways. VEGF concentration is highest near the middle of hypoxic regions, and growing sprouts then remain and meander in such regions, rather than connecting with other vessels. Therefore, this effect was excluded.

### Branching angles and tension-induced migration

**Figure 3:**
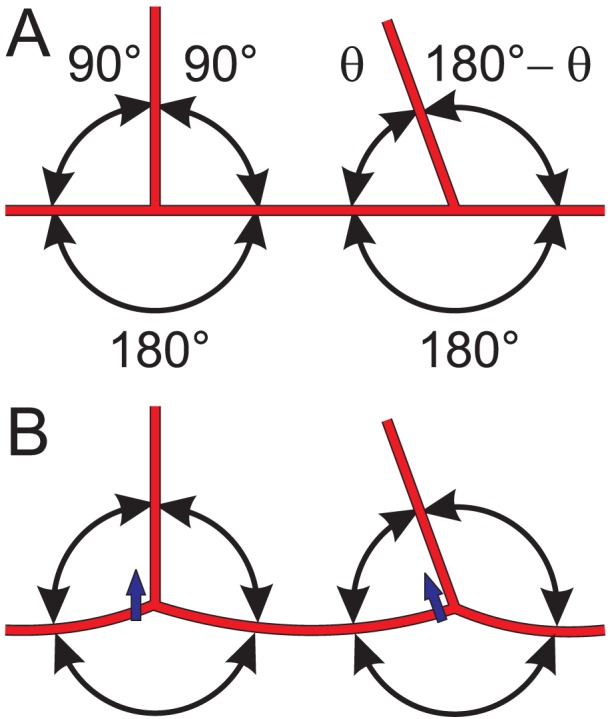
Bifurcation angles resulting from vessel sprouting and connection. (**A**) A sprout at 90° to the parent vessel creates initial bifurcation angles of 90°, 90° and 180°. A sprout forming a connection to an existing vessel creates initial bifurcation angles of θ, 180°−θ and 180°, where θ depends on the direction from which the sprout approaches. (**B**) Migration through the tissue (short blue arrows) driven by tension in each segment and the resulting imbalance of forces acting within the network results in a reduction of the 180° bifurcation angle and increases in the other two angles.

In the model, new branch points are formed by sprouting from existing segments, and by coalescence of sprouts with existing segments. Of the three branching angles formed by such events, one is necessarily 180° and the other two must average to 90° (Figure 3A). This would still be the case even if the model was modified to include the effect of chemical cues on sprouting direction [42], such that sprouts formed at variable angles to the parent vessel. If no mechanism for change of branch angles is included, the resulting distribution of branching angles has peaks at 90° and 180°. In the observed network (see Results), the angles are smoothly distributed about the mean (120°). This discrepancy in angle distribution implies that branching angles must change and vessels must migrate through tissue after formation of bifurcations (Figure 3B), by a mechanism that has not previously been described.

Blood vessels *in vivo* are normally subject to longitudinal tension [43], [44]. Structural components of the interstitial space, including collagen, are subject to continuous turnover in normal tissues [45]. These observations suggest a potential mechanism for remodeling of branch angles, in which the net forces on each segment resulting from axial tension tend to pull it through the interstitium, and movement is made possible by the continuous dissolution and synthesis of collagen fibers.

This mechanism is implemented in the model as follows. Each node (including non-branching nodes) migrates through the surrounding tissue at a rate dependent on the resultant force due to vessel tensions, which are assumed proportional to diameter. The normalized force is
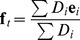
(14)where the sum is over the segments at the node, *D_i_* is diameter and **e** *_i_* is a unit vector parallel to the segment. If |**f** *_t_*| exceeds a threshold *λ_t_*, the node migrates in the direction of **f** *_t_* with velocity proportional to |**f** *_t_*|−*λ_t_*, i.e.
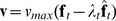
(15)where *v_max_* is the maximum speed and 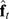 is a unit vector in the direction of **f** *_t_*. Inclusion of the threshold stabilizes curved vessels which otherwise would eventually straighten. Chosen values of *v_max_* and *λ_t_* yield curvatures comparable to those observed. In this model for tension-induced migration, total vessel length decreases slowly in the absence of sprouting, until the normalized force at each node approaches the threshold value.

### Structural adaptation and pruning

The model for structural adaptation of flowing segments was developed previously [19], [20], and is used here with slight modifications. The diameter *D* of each segment varies in response to several stimuli:
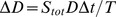
(16)where Δ*t* is the time step and *T* is the timescale [46]. The total signal is
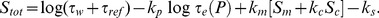
(17)


The first two terms represent responses to wall shear stress *τ_w_* (in dyn/cm^2^) and intravascular pressure *P* (in mmHg). The function
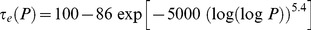
(18)describes the correlation of *τ_w_* (in dyn/cm^2^) with *P* [20]. A metabolic signal is generated in each vessel dependent on vessel *P_O_*_2_,
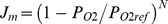
(19)where *N* sets the oxygen sensitivity. Previously [20] *N* = 1 was assumed, but in the present simulations this leads to loss of many vessels needed for adequate oxygen supply, because the signal lacks sensitivity to *P_O_*_2_ at low levels. Here *N* = 2 is assumed. Downstream transmission of the metabolic signal is modeled by a convective flux generated in each segment in proportion to *J_m_l_seg_* and accumulated downstream, where *l_seg_* is segment length. The local metabolic signal is
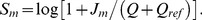
(20)


Each segment contributes to the conducted response *J_c_* in proportion to *S_m_l_seg_*. (Previously [20], the factor *l_seg_* was omitted.) Conducted responses travel upstream, decaying as exp(−*s*/*L_c_*), where *s* is distance. At converging bifurcations relative to direction of conduction, incoming signals are summed. At diverging bifurcations, the signal is divided equally among the upstream vessels. The conducted metabolic signal is
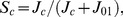
(21)where the magnitude of *J*_01_ is set to allow dropout of short shunt pathways while retaining longer, functional pathways. The shrinking tendency *k_s_* was adjusted to give a flow rate 36.9 nl/min at oxygen demand 2 cm^3^O_2_/100 cm^3^/min, matching the observed network. A random component, normally distributed with zero mean and standard deviation *Ran-k_s_* is included in *k_s_* [47]. If a diameter drops below 3 µm, the minimum for passage of red blood cells, the segment is pruned, as are any other segments whose flow ceases as a result.

## Results

**Figure 4:**
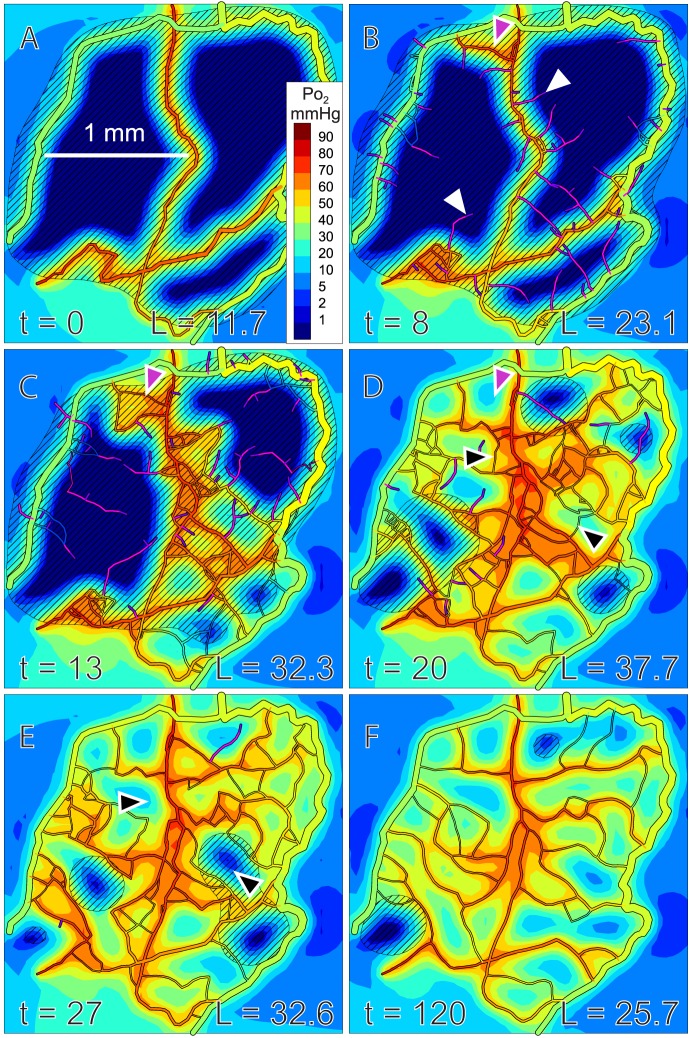
Simulated angiogenesis, showing oxygen and VEGF distributions. Oxygen levels in vessels and tissue are color coded according to scale at right. Dark blue indicates hypoxic tissue. Diagonal shading indicates VEGF above threshold concentration. Time (*t*) in days and total vessel length (*L*) in mm are indicated. Maximal rate of sprout formation is 2 mm^−1^day^−1^. Tissue oxygen demand is 2 cm^3^/100 cm^3^/min. (**A**) Initial configuration. (**B**) Sprouts (white arrows) and a short flow pathway between arterioles and venules (a-v shunt, purple arrow) are generated. (**C**) More complex flow pathways form, leading to improved oxygenation (lower right region). (**D**) Improved oxygenation leads to decreased VEGF levels. Structural adaptation causes pruning of the a-v shunt (purple arrow), but some redundant flow pathways remain (black arrows). Total vessel length reaches its maximum. (**E**) Structural adaptation leads to pruning of redundant flow pathways (black arrows). (**F**) Final refined network. Virtually no hypoxia remains and VEGF levels are generally below threshold. See online supplement, Video S1, for movie clip.

Results of a simulation are shown in Figure 4. Initially, sprouts grow and connect to form dense mesh-like structures, which are refined to produce more hierarchical structures with an orderly progression of vessel diameters. In this process, redundant segments are removed, including those forming very short shunt pathways (Figure 4C–D). While the remaining flow pathways show widely varying lengths, the diameters of the short flow pathways are relatively small, such that they do not draw much flow away from the longer flow pathways. Total vessel length peaks at about 20 days, and declines towards a stable steady state, in a temporal sequence similar to that observed in wound healing [48].

**Figure 5:**
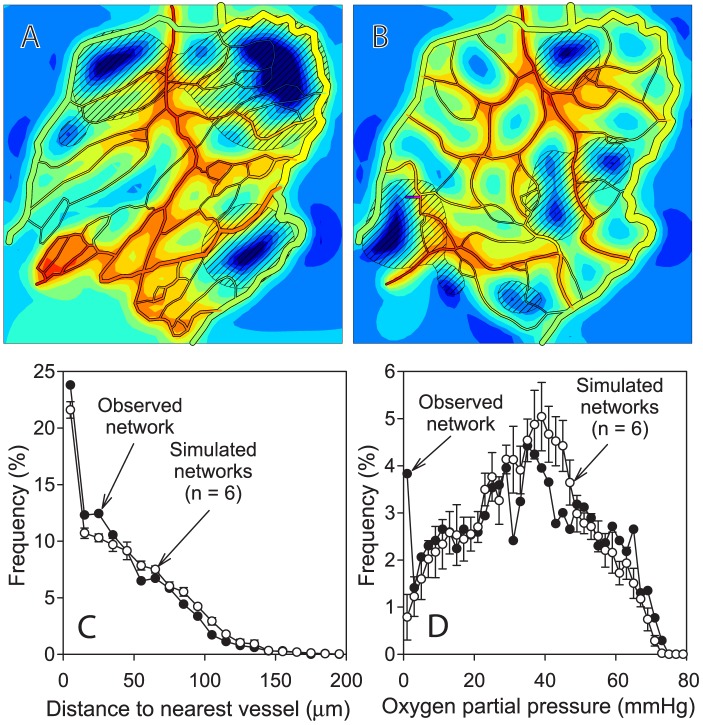
Comparison of simulated and observed network characteristics. Simulated oxygen and VEGF distributions in experimentally observed network (**A**), and in simulated angiogenesis at *t* = 200 days (**B**). The observed network structure is derived from the image shown in Figure 2A. The network at *t* = 200 days was derived from the same initial network as shown in Figure 4A but with a different seed for random number generation. Oxygen demand, rate of sprout formation, length scale, color coding of oxygen levels and diagonal shading indicating VEGF above threshold are as in Figure 4. (**C**) Frequency distribution of distance of tissue points to nearest vessel. (**D**) Frequency distribution of oxygen levels at tissue points. Results for simulated networks are mean ± standard deviation for n = 6 simulations with different seeds for random number generation.

Characteristics of simulated networks are compared in Table 2 and Figure 5 with those of the experimentally observed network from which the initial configuration (Figure 4A) was derived. The simulated networks are qualitatively similar to the observed network and values of key parameters are comparable. The simulated networks have slightly lower total vessel length than the observed network, and the distribution of distance from tissue points to the nearest vessel is slightly right skewed in the simulated networks relative to the observed network. Despite the lower vascular density, the mean tissue *P_O_*_2_ in the simulated networks agrees closely with that in the observed network. Moreover, the simulated networks have a narrower distribution of *P_O_*_2_ and less hypoxic tissue (*P_O_*_2_<1 mmHg). These results imply that the assumed mechanisms of angiogenesis and adaptation can generate network structures that match and indeed slightly exceed the performance of the observed network with regard to oxygen transport.

**Table 2:** Comparison of simulated networks with experimentally observed network.

	Observed network	Simulated networks: mean ± s.d. (n = 6)
Total vessel length (mm)	30.2	26.1±0.92
Mean distance of tissue points to nearest vessel (µm)	39.3	44.7±1.45
Flow rate (nl/min)	36.9	34.9±2.75
Mean tissue *P_O_*_2_ (mmHg)	34.4	35.2±1.14
Hypoxic fraction (%)	3.24	0.355±0.308

**Figure 6:**
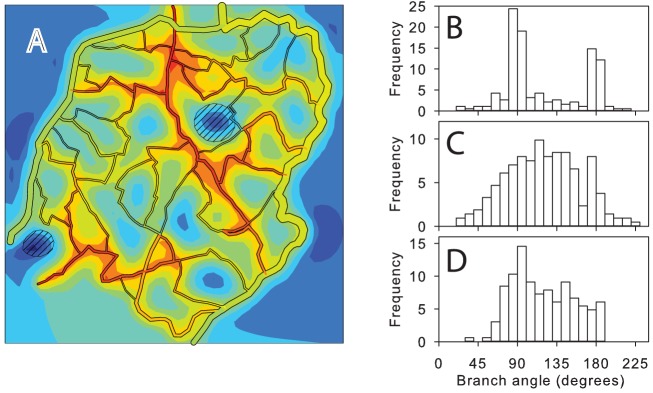
Effect of tension-induced vessel migration on distribution of branching angles. (**A**) Example of network structure generated when tension-induced migration is suppressed, at *t* = 200 days. Other conditions are as in Figure 4. (**B**) Distribution of branching angles for network shown in A. (**C**) Distribution of branching angles for observed network structure. (**D**) Distribution of branching angles for network shown in Figure 4 at *t* = 200 days, including tension-induced migration.

The effect of tension-induced lateral migration of vessels on the distribution of branching angles is illustrated in Figure 6. If no mechanism for change of branch angles is included, the resulting network structures have many vessels with abrupt changes in direction (Figure 6A) and the distribution of branching angles has peaks at 90° and 180° (Figure 6B). Inclusion of tension-induced migration in the model results in a more uniform distribution (Figure 6C) although the distribution is not as broad as that obtained from the experimentally observed network (Figure 6D).

**Figure 7:**
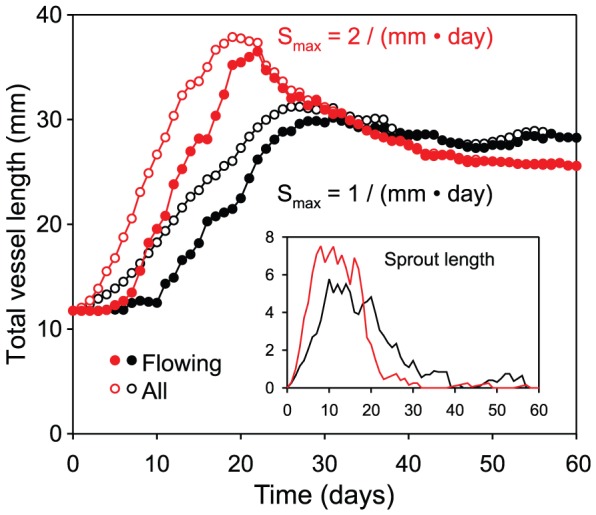
Dynamics of network generation. Variation of total vessel length during an individual simulation of angiogenesis. Main plot: vessel length. Inset: length of non-flowing sprouts. Initially, total length increases rapidly due to sprouting. After sprouts form connections, flowing length increases. When sprout formation rate is 2 mm^−1^day^−1^ (red curves), summed vessel length shows a strong peak at 20 days and then decreases, stabilizing after 50 days. For reduced sprout formation rate (1 mm^−1^day^−1^, black curves), peak vascular length is reduced, stabilization takes longer and total vessel length remains higher.

The variation of total vessel length during simulated angiogenesis is shown in Figure 7. When the maximal rate of sprout formation is 2 mm^−1^day^−1^, as in Figures 4 and 5, an overshoot in vascular density is predicted. With a lower rate of sprout formation, 1 mm^−1^day^−1^, the overshoot is smaller and the network takes longer to stabilize, remaining at a higher vascular density. Over-abundant initial production of vessels is therefore needed for efficient vascular network generation. If the mesh structure generated during the initial phase of angiogenesis is not sufficiently dense, it is inadequate to meet oxygen needs and further sprout formation is stimulated, prolonging the network's instability.

**Figure 8:**
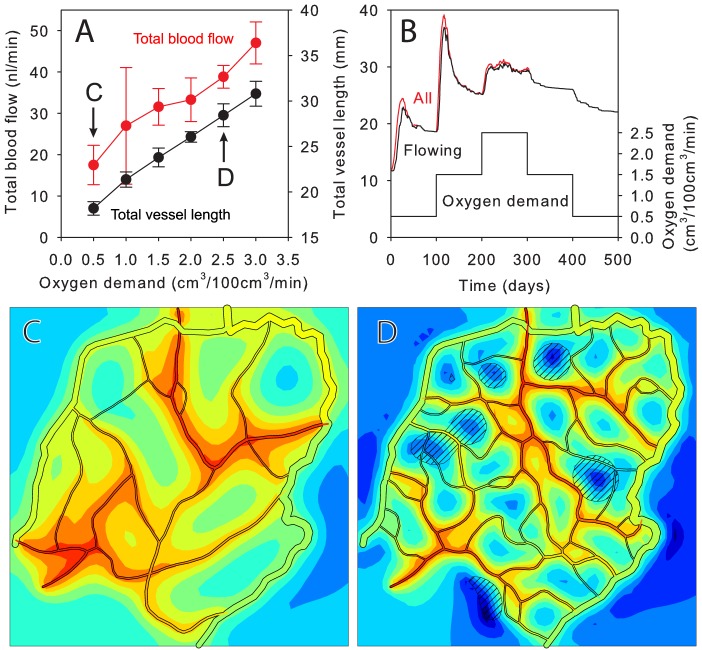
Variation of network properties with oxygen demand. (**A**) Total blood flow and vessel length at *t* = 200 days, for fixed arteriole-venule pressure drop (mean ± s.d. for 6–12 runs). (**B**) Time-dependent response of total vessel length to step changes in demand. Lower curve: oxygen demand. Upper curves: total vessel length (flowing, all, mean of 3 runs). (**C, D**) Network structures for demand 0.5 and 2.5 cm^3^/100 cm^3^/min. Length and color scales as in Figure 4.

The assumed mechanisms allow adaptation to changing conditions. Effects of varying oxygen demand were explored, assuming a fixed arteriole-venule pressure drop. With increasing demand, total vessel length and flow rate increased (Figure 8A). Resulting network structures are shown in Figures 8C and D. Figure 8B illustrates the dynamic response of the network to changes in oxygen demand over time. A step increase (in cm^3^/100 cm^3^/min) from 0.5 to 1.5 stimulates an overshoot in total vessel length followed by stabilization. After a step increase to 2.5, the rate of sprout formation is not sufficient to produce an overshoot and stability is not achieved. This result with a sprout rate of 2 mm^−1^day^−1^ is similar to the behavior at an oxygen demand of 2 and a sprout rate of 1 mm^−1^day^−1^ shown in Figure 7, suggesting that the rate of sprout formation needed for efficient network generation is sensitive to oxygen demand. With step decreases in demand, the network regresses, but the vessel length remains higher than before at the same demand. This suggests that a period of high demand (e.g. exercise training) can lead to a long-term increase in vascular density.

**Figure 9:**
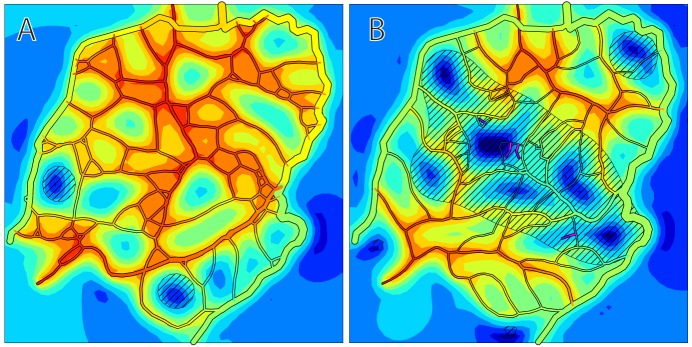
Effects of inhibiting adaptation or conducted responses. (**A**) Angiogenesis without structural adaptation and pruning. Resulting structure is mesh-like, without hierarchical structure. (**B**) Simulation of angiogenesis with reduced conducted response strength. Short flow pathways from feeding to draining vessels carry most of the flow. Regions remote from feeding vessels receive inadequate perfusion. Length and color scales as in Figure 4.

Further simulations were used to explore the effects of inhibiting specific biological patterning mechanisms (Figure 9). Without structural adaptation and pruning, all new vessels remain at their initial diameter of 10 µm. A stable vessel network is formed, but the total vessel length is higher (31.8 mm) than in the simulated normal case (25.6 mm). Instead of a hierarchical branching pattern, a mesh-like structure develops. If conducted responses are inhibited by reducing the coefficient of the conducted response from 2.45 to 0.5, the network does not achieve a stable, well-oxygenated state. Functional shunts between arterioles and venules are not suppressed [21], and only regions close to the feeding arterioles receive adequate oxygenation.

## Discussion

These results show that the combination of stochastic angiogenesis stimulated by a growth factor, structural adaptation and pruning in response to hemodynamic and metabolic stimuli is capable of solving the ‘problem’ of vascular patterning and can generate hierarchical networks with low diffusion distances. To establish and maintain such networks, the following mechanisms are essential:

- generation of a diffusible vessel growth factor in hypoxic tissue regions;
- formation of vessel sprouts in response to above-threshold levels of growth factor;
- maintenance of sprouts without pruning before they connect to other vessels;
- ability of sprouts to connect with other vessels forming patent flow pathways;
- diameter adaptation of flowing vessels to hemodynamic and metabolic stimuli and upstream conducted responses;
- elimination of redundant vessels by pruning.

The initial network, derived by reducing an observed mesenteric network to a minimal ‘skeleton’, allowed testing of the model by comparing predicted structures with the actual network. Initial conditions for angiogenesis in development, wounds, exercise or tumor growth may differ from those assumed here. In most tissues, networks ramify in three dimensions. While simulations of other tissues may reveal the need for additional mechanisms or constraints for formation of realistic network structures, the arguments leading to the above conclusions are not specific to the assumed geometry.

The ability of a vessel to form a sprout or to connect with a sprout is here assumed independent of vessel type (arterial or venous). While arterioles and venules show different expression of genes involved in angiogenesis [49], both types participate in angiogenesis, and arterial-venous plasticity is observed during neovascularization [50].

The mechanisms of angiogenesis are more numerous and complex than those included here. Multiple growth factors participate in the control of sprouting angiogenesis. VEGF exists in several isoforms, and is not the only factor involved. Other factors may play equally important roles. Nonetheless, it can be concluded from our results that angiogenesis in response to a growth factor released in hypoxia can result in vascular patterns that are consistent with *in vivo* observations. Our approach demonstrates a minimal set of mechanisms that is sufficient to solve the vascular patterning problem, generating structures that combine low diffusion distances to all tissue cells with hierarchical branching, and adapt to changing conditions. The model allows assessment of the roles of individual mechanisms in the patterning process and changes resulting from their modification. It shows that network formation involves closely coupled processes of angiogenesis, structural adaptation and pruning. Resulting insights may stimulate further experimental investigations of angiogenesis and development of novel therapeutic approaches.

## Supporting Information

Video S1 **Simulated angiogenesis, showing oxygen and VEGF distributions.** Oxygen levels in vessels and tissue are color-coded according to scale at right in mmHg. Dark blue indicates hypoxic tissue. Diagonal shading indicates VEGF above threshold concentration. Time (*t*) in days is indicated on each frame. See Figure 3 caption for further description of this simulation.
(AVI)
